# Bilateral lower-limb asymmetry and phase-specific trunk acceleration during the Timed Up and Go in older women: an analysis based on muscle morphology and antagonist coactivation

**DOI:** 10.3389/fphys.2026.1915589

**Published:** 2026-10-02

**Authors:** Danming Xu, Hongqin Xu, Xin Zhang, Ming Li, Xiangwei Meng

**Affiliations:** 1 School of Art and Sciences, Chengdu College of University of Electronic Science and Technology of China, Chengdu, China; 2 School of Sports and Health, Guizhou Medical University, Guiyang, China; 3 College of Education and Sports Sciences, Yangtze University, Jingzhou, China

**Keywords:** antagonist coactivation, fall risk, muscle thickness, root mean square of acceleration, Timed Up and Go

## Abstract

The Timed Up and Go (TUG) test is widely used to assess mobility and fall risk in older adults, but total completion time does not capture phase-specific motor control. This study quantified bilateral lower-limb asymmetry in muscle morphology and neuromuscular control and examined its association with phase-specific trunk acceleration during the TUG. Twenty-seven healthy older women (78.96 ± 2.44 years) participated. Bilateral muscle thickness was measured by B-mode ultrasound, and bilateral surface electromyography and L4 trunk acceleration were recorded during a five-phase instrumented TUG. Mean and peak coactivation index (CCI) and asymmetry index (ASI) of the knee (QUAD-BF) and ankle (TA-MG) muscle pairs, together with anteroposterior and mediolateral trunk acceleration RMS (RMS_AP_, RMS_ML_), were calculated. No significant group-level left–right differences in muscle thickness were detected, although ASI exceeded 15% for all four muscles. After FDR correction, significant bilateral differences were retained for mean CCI_QUAD-BF_ during sit-to-stand (p< 0.001, q = 0.005), mean CCI_TA-MG_ during sit-to-stand (p = 0.001, q = 0.013) and peak CCI_QUAD-BF_ during sit-to-stand (p = 0.002, q = 0.013); the difference for mean CCI_QUAD-BF_ during return walk (p = 0.016) did not survive correction. Coactivation ASI was generally higher at the knee than the ankle. After Benjamini–Hochberg false discovery rate correction, three peak coactivation asymmetry correlations with RMS_AP_ survived: peak ASI_TA-MG_ during outbound walk (r = 0.72, q< 0.001), peak ASI_QUAD-BF_ during turn (r = 0.53, q = 0.043) and peak ASI_TA-MG_ during sit-to-sit (r = 0.57, q = 0.044). The negative correlations of ASI_MG_ with RMS_AP_ during sit-to-stand and outbound walk (r = −0.52, r = −0.51) did not survive FDR correction. No ASI correlated significantly with RMS_ML_. Bilateral asymmetry was heterogeneous in direction, and phase-specific peak coactivation asymmetry was more consistently associated with anteroposterior trunk acceleration than muscle thickness. These measures may provide complementary information beyond muscle morphology or total TUG time, but their reliability and clinical relevance require further investigation.

## 1 Introduction

Falls are one of the leading causes of disability, loss of independence and death among community-dwelling older adults. Around 30% of community-dwelling older adults experience at least one fall each year, and this proportion rises steadily with age (Li et al., 2023). Among the many risk factors for falls, mobility-related factors such as balance, gait, muscle strength and mobility function account for the largest share (Saunders et al., 2025). Early and sensitive assessment of mobility is therefore a key element of fall prevention (Montero-Odasso et al., 2022). Of the available tools, the Timed Up and Go (TUG) test is widely used to screen mobility and fall risk in older adults because it is simple, quick and requires no specialized equipment (Podsiadlo and Richardson, 1991).

The conventional TUG uses total completion time as its single outcome. However, growing evidence indicates that total time is of limited sensitivity in higher-functioning older adults and cannot reveal the subtle differences in motor control that occur during standing up, walking, turning and sitting down (Salarian et al., 2010; Weiss et al., 2010). To address this limitation, the instrumented TUG uses a trunk-mounted inertial sensor to extract phase-specific kinematic and stability measures from the acceleration signal, capturing dynamic control features that total time cannot reflect (Salarian et al., 2010; Zampieri et al., 2010). Among these measures, the root mean square (RMS) of trunk acceleration quantifies the magnitude of trunk acceleration fluctuations, which may reflect postural control, propulsion, braking and speed changes. Although RMS does not directly measure postural stability, it has been used as a proxy for dynamic control during walking in older adults (Menz et al., 2003). The different directions carry different functional meaning: the anteroposterior (AP) component is more strongly influenced by propulsion, braking and changes in speed, whereas the mediolateral (ML) component is associated primarily with step-by-step foot placement and lateral balance control (MacKinnon and Winter, 1993; Menz et al., 2003). Standing up, deceleration during turning and braking when sitting down all involve rapid adjustments of forward momentum. Examining trunk acceleration by direction and by phase therefore allows a more detailed characterization of trunk acceleration during functional tasks in older adults.

Trunk acceleration during functional tasks is influenced by many factors, among which bilateral lower-limb asymmetry warrants particular attention. Older adults commonly show varying degrees of inter-limb asymmetry in muscle mass, strength and power, and strength asymmetry has been linked to greater gait asymmetry and variability (LaRoche et al., 2012; Mertz et al., 2019). At the group level, however, there is often no fixed dominant side, and the direction of asymmetry is highly heterogeneous between individuals (Sadeghi et al., 2000). Most previous studies have characterized asymmetry using resting structural measures such as muscle strength or morphology. Yet these measures mainly reflect force-generating potential and may not directly capture bilateral control during complex dynamic tasks. Examining lower-limb asymmetry at the neuromuscular control level, in addition to the morphological level, may therefore offer a perspective that is more closely tied to function.

At the neuromuscular control level, antagonist coactivation is an important indicator of joint stability and control strategy. With ageing, older adults often increase lower-limb antagonist coactivation to raise joint stiffness and to compensate for declining strength and impaired balance, a pattern regarded as a neuromuscular defense strategy (Hortobágyi and DeVita, 2000; Iwamoto et al., 2017; Acuña et al., 2019). While coactivation enhances joint stability, it also increases energy cost and may limit rapid postural adjustment (Latash, 2018). Notably, previous research on lower-limb coactivation in older adults has focused mainly on a single limb. For example, one study of older adults walking on a compliant surface found greater agonist–antagonist coactivation at the ankle and knee than in younger adults, accompanied by a redistribution of propulsive demand towards more proximal muscles (Jeon et al., 2023), and recent work has continued to characterize the neuromuscular control of the ankle in older adults from a single-limb perspective (Yaguchi-Horiuchi et al., 2025). These studies, however, have analyzed coactivation on one side only. Whether coactivation is balanced between the left and right limbs, whether this bilateral asymmetry varies across TUG phases, and how it relates to dynamic stability remain unclear. To date, no study has examined how bilateral antagonist coactivation asymmetry varies across individual TUG phases or how it relates to phase-specific trunk acceleration. These questions are particularly relevant in the TUG, a task dominated by non-steady phases that place high demands on bilateral coordination.

Accordingly, this study examined healthy community-dwelling older women using simultaneously recorded bilateral surface electromyography and L4 trunk acceleration. We quantified bilateral lower-limb asymmetry at two levels, muscle morphology (thickness) and neuromuscular control (antagonist coactivation), and divided the TUG into five phases—sit-to-stand, outbound walk, turn, return walk and sit-to-sit—to examine the phase-specific relationship between asymmetry and the root mean square of trunk acceleration in the anteroposterior and mediolateral directions (RMS_AP_, RMS_ML_). We hypothesised that (1) bilateral lower-limb asymmetry would be common but heterogeneous in direction; (2) greater peak coactivation ASI would be positively associated with RMS_AP_, reflecting larger anteroposterior trunk acceleration; and (3) this association would be stronger for coactivation ASI than for muscle thickness ASI and would vary by TUG phase and acceleration direction.

## 2 Methods

### 2.1 Participants

Twenty-seven healthy community-dwelling older women volunteered for this study (age 78.96 ± 2.44 years; height 149.72 ± 4.38 cm; body mass 54.68 ± 8.82 kg; BMI 24.40 ± 3.75 kg/m²). Participants were recruited through local senior activity centers and public health service programs, and all were community residents who lived independently and walked unaided. The inclusion criteria were: (1) no history of serious cardiovascular, musculoskeletal or neurological disease; (2) ability to understand the test instructions and to complete the informed consent process independently; (3) no known or self-reported neuromuscular impairment likely to affect balance or gait; (4) no history of walking difficulty, dizziness or balance disorder; and (5) ability to walk continuously at a normal pace for at least 20 min without assistance or rest. The sample size was estimated by an *a priori* power analysis (G*Power 3.1.9.7) for a bivariate correlation, with reference to LaRoche et al. (2012). Assuming ρ = 0.5 for the association between peak coactivation ASI and trunk acceleration RMS as the primary analysis, α = 0.05 and power (1 − β) = 0.8, a minimum of 21 participants was required; the enrolled sample of 27 satisfied this requirement. Before testing, the procedures and potential risks were explained in detail to all participants, who then gave written informed consent. The study protocol complied with the Declaration of Helsinki and was approved by the Ethics Committee of Guizhou Medical University (approval number: 202596).

### 2.2 Muscle thickness measurement

Because this study aimed to quantify absolute bilateral asymmetry rather than to compare a predetermined dominant with a non-dominant limb, all bilateral measurements—including ultrasound thickness and surface electromyography—were assigned by anatomical side (left versus right) throughout. Limb dominance was not formally assessed. Muscle thickness was measured following the method of Li et al. (2026), using an Esaote MyLab™ Alpha ultrasound system (Esaote, Genoa, Italy) fitted with an L4–15 linear probe (4–15 MHz). Real-time B-mode imaging was used to acquire images of four muscles: the RF, BF, TA and MG. All images were acquired under uniform settings (frequency 8–12 MHz; gain 80 dB; dynamic range 110 dB; scanning depth 52 mm), with focal depth adjusted individually to the position of each target muscle to optimize image quality. To minimize inter-operator variability, all measurements were performed by the same experienced operator. Before measurement, participants sat quietly for 5 min to allow the target muscles to relax. Standardized positions were used for each muscle: a supine position with the knee naturally extended for the RF and TA, and a prone position for the BF and MG. In both positions, the feet hung freely over the end of the examination table to avoid additional muscle tension from foot support that could affect the measurement. The measurement site for each muscle was determined using standardized anatomical landmarks: for the RF, at 50% of the distance from the anterior superior iliac spine to the superior border of the patella along the anterior midline of the thigh; for the BF, at 50% of the distance from the ischial tuberosity to the lateral epicondyle of the femur along the posterior midline of the thigh; for the TA, at the proximal one-third of the distance from the inferior border of the tibial tuberosity to the tip of the lateral malleolus along the anterolateral aspect of the leg; and for the MG, at approximately 30% of the distance from the popliteal crease to the calcaneal tuberosity, corresponding to the level of maximum calf circumference. At each site, the operator confirmed the location of maximum cross-section with a preliminary longitudinal sweep and marked it on the skin with a pen. The same landmarks and procedures were used bilaterally. A generous amount of water-soluble coupling gel was then applied to the probe surface, which was placed perpendicular to the marked point with only the minimal pressure needed to ensure acoustic contact, so as to avoid muscle deformation from compression. Three static images were acquired consecutively at each site, and image quality was checked in real time to ensure that the superficial and deep fascial borders were clearly distinguishable.

### 2.3 Test preparation

To capture kinematic and neuromuscular information simultaneously during the TUG, several acquisition systems were started in parallel. Trunk kinematics were recorded with a triaxial accelerometer (Zephyr BioHarness, Zephyr Technology, Annapolis, MD, USA) at 100 Hz, secured at the level of the fourth lumbar (L4) spinous process with an elastic strap. Surface electromyography (sEMG) was recorded synchronously at 2000 Hz using a Noraxon Ultium-ESP wireless system (Noraxon USA, Inc., Scottsdale, AZ, USA) from the RF, VM, VL, BF, TA and MG bilaterally. Before electrode placement, the skin over each muscle belly was prepared in a standardized manner (shaving and cleaning with alcohol to remove surface oils) to reduce skin–electrode impedance. A pair of Ag/AgCl bipolar electrodes (Ambu^®^ BlueSensor N; inter-electrode distance 1 cm) was then attached over the muscle belly along the direction of the muscle fibers, with the reference electrode placed over the tibial tuberosity. All electrodes and sensors were reinforced with athletic tape to reduce motion artefacts. The system amplifier had an input impedance of >1000 MΩ and a common-mode rejection ratio (CMRR) of >100 dB. Electrode placement and skin preparation followed the SENIAM recommendations for surface electromyography (Hermens et al., 2000).

### 2.4 TUG test

Functional mobility was assessed using the TUG proposed by Podsiadlo and Richardson (1991). Participants sat upright on a stable, armless chair with their feet flat on the floor and their hands resting on their thighs. On the command “go”, each participant stood up, walked in a straight line at a self-selected comfortable speed for 3 m to a floor marker, turned around a marker cone, returned along the same path and sat down again, without using any walking aid. Before the formal trials, the complete procedure was explained and demonstrated by the same researcher, and participants completed one or two familiarization trials. After a 5 min rest, they performed three formal trials, with a 3 min rest between trials to avoid cumulative fatigue. The same researcher gave the commands and supervised task performance throughout to ensure consistent test conditions.

### 2.5 MVIC test

To normalize the sEMG amplitude during the TUG, maximal voluntary isometric contraction (MVIC) tests were performed for the RF, VM, VL, BF, TA and MG bilaterally after the TUG. MVIC was placed after the functional task to avoid any fatigue from maximal contractions affecting TUG performance, with sufficient rest between the two tests to ensure recovery. In standardized positions for each muscle, participants performed a maximal contraction against manual resistance applied by the tester, each lasting 5 s and repeated three times per muscle, with 60–90 s of rest between trials; consistent verbal encouragement was given throughout to ensure maximal effort. All MVIC tests were administered by the same examiner using identical procedures for both limbs to minimize inter-examiner variability and potential side-to-side normalization bias. The test tasks and positions were as follows: resisted knee extension in a seated position with the hip and knee flexed to about 90° for the RF, VM and VL; resisted knee flexion in a prone position for the BF; ankle dorsiflexion with slight inversion for the TA; and resisted ankle plantarflexion for the MG. Before each MVIC, the movement was explained and demonstrated, and participants first performed a submaximal practice contraction to become familiar with the task.

### 2.6 Data processing

All data processing and outcome calculations were performed with custom scripts written in Python 3.13 (PyCharm 2024.1 Professional Edition; JetBrains, Prague, Czech Republic). Ultrasound images were processed following the method of Zhang et al. (2025), with semi-automatic analysis of the original bitmap images (860 × 808 pixels). All images were acquired at a fixed scanning depth (D = 52 mm) with identical instrument settings. The spatial calibration was determined from the on-screen depth scale: the distance between the 0 cm and 4 cm markers spanned 415 pixels in the axial direction, yielding a calibration factor of 103.75 pixels/cm (1 pixel = 0.0964 mm). Muscle thickness was measured in pixels and converted to millimeters using this factor. Pre-processing comprised grayscale conversion, contrast-limited adaptive histogram equalization (CLAHE), Gaussian blurring for noise reduction and edge detection, in order to highlight the fascial borders. The analyst first defined a template region of interest (ROI) on the image and matched it to the three repeated images of the same muscle by two-dimensional cross-correlation. The superficial and deep fascial positions were then identified manually, and the mean muscle thickness of each image was calculated as the perpendicular distance at three points within the ROI.

The processing of kinematic and EMG signals, the identification of TUG events and the calculation of outcomes were carried out as follows. Raw signals were first digitally filtered. Trunk acceleration was denoised with a fourth-order, zero-phase Butterworth low-pass filter with a cut-off frequency of 20 Hz, and the sEMG (using the same settings for the MVIC and TUG tasks) was band-pass filtered at 20–500 Hz with a fourth-order, zero-phase Butterworth filter to remove motion artefacts and high-frequency noise. The sEMG amplitude was normalized as follows: the RMS of the filtered signal was computed over a 100 ms sliding window; for each muscle and each side, the maximum of the 100 ms sliding RMS across the three MVIC trials was taken as the normalization reference for that muscle; and the EMG RMS at each time point during the TUG was divided by the corresponding MVIC reference and multiplied by 100 to give the percentage of maximal voluntary isometric contraction (%MVIC). The TUG phases were identified using a custom rule-based algorithm adapted from the instrumented-TUG approaches reported by Salarian et al. (2010); Zampieri et al. (2010), and Weiss et al. (2010), combining the characteristics of the L4 triaxial acceleration signal with the fixed temporal sequence of the TUG actions. The triaxial acceleration was first low-pass filtered to 2–3 Hz, and the low-frequency gravitational component was subtracted to obtain the dynamic acceleration. The 0.5 s sliding RMS of the dynamic resultant acceleration was then computed and used to identify the main movement peak of the sit-to-stand phase and the relatively stable point that followed it. The interval from the end of sit-to-stand to the start of the turn was defined as the outbound walk; the turn was determined from a turning feature signal derived from the rate of change of the triaxial acceleration, together with the temporal constraint that the turn occurs in the middle of the TUG; the interval from the end of the turn to the start of sitting down was defined as the return walk; and the onset of the marked movement change in the final part of the test was defined as the start of the sit-to-sit phase. Each TUG was thus divided into five phases—sit-to-stand, outbound walk, turn, return walk, and sit-to-sit—and all automatic segmentations were visually checked for quality against the acceleration and sEMG waveforms.

Using the valid data from each TUG phase, RMS_AP_ and RMS_ML_ at L4 were calculated to quantify trunk acceleration fluctuations in the respective directions. To assess the level of antagonist coactivation, the coactivation index (CCI) was computed from the %MVIC-normalized 100 ms sliding RMS for two muscle pairs: quadriceps–biceps femoris (QUAD-BF) and tibialis anterior–medial gastrocnemius (TA-MG). The quadriceps signal was obtained as the mean of the normalized RMS of the RF, VM and VL at each time point, whereas the BF, TA and MG were represented by the normalized RMS of each individual muscle. Following the method reported by Gagnat, Brændvik and Roeleveld (2020), the CCI at each time point was calculated as:


$$CCI\left(t\right)=200\times \frac{\min\left[A\left(t\right),B\left(t\right)\right]}{A\left(t\right)+B\left(t\right)}$$

where A(t) and B(t) are the %MVIC-normalized 100 ms sliding RMS of the two muscle groups at time point t. The CCI ranges from 0 to 100%, where 0% indicates activation of only one muscle group and 100% indicates equal activation of the two groups. The mean and maximum of the instantaneous CCI series were calculated for each TUG phase to give the mean CCI and the peak CCI. It should be noted that the CCI reflects the relative overlap of activation between the two muscle groups rather than their absolute activation level.

The coactivation asymmetry index was calculated from the mean CCI and the peak CCI of each TUG phase for the left and right sides, yielding the mean CCI asymmetry index and the peak CCI asymmetry index (ASI):


$$ASI\left(\%\right)=\frac{200\times \left|X_{R}-X_{L}\right|}{X_{R}+X_{L}}$$

where X_R_ and X_L_ are the mean or peak CCI of the right and left sides, respectively. The ASI has a theoretical range of 0–200%, where 0% indicates perfect left–right symmetry and higher values indicate greater asymmetry. The same formula was used to calculate the asymmetry index of bilateral muscle thickness.

For each participant, every phase-level outcome variable—including mean CCI and peak CCI for both muscle pairs on both sides, the corresponding ASI values, and RMS_AP_ and RMS_ML_—was first computed separately for each of the three TUG trials. We assessed the repeatability of these measurements before averaging the three trials for subsequent statistical analysis. For ultrasound thickness, within-session imaging precision was assessed using the coefficient of variation (CV) across the three images of each muscle. For the TUG, between-trial consistency was assessed using the CV across the three trials for RMS_AP_, RMS_ML_, and the mean CCI of each muscle pair in each phase. In addition, test–retest reliability across the three complete TUG trials was assessed for RMS_AP_ and RMS_ML_, with good to excellent reliability observed for RMS_AP_ (ICC(A,3) = 0.87, 95% CI [0.68, 0.95]) and RMS_ML_ (ICC(A,3) = 0.96, 95% CI [0.91, 0.98]). Phase-specific ICCs were not calculated for the CCI, ASI, RMS_AP_, or RMS_ML_ outcomes; therefore, reliability at the individual TUG-phase level was not established. The mean CV of all assessed measures was below 15%, indicating acceptable repeatability. Based on these results, the three images or trials were averaged for each outcome variable, and the resulting mean values were used in subsequent statistical analysis.

### 2.7 Statistical analysis

All statistical analyses were performed in R 4.3.2 (R Foundation for Statistical Computing, Vienna, Austria). Continuous variables are presented as mean ± SD. The normality of each variable was assessed using the Shapiro–Wilk test together with Q–Q plots, which were also used to inspect the data distribution and potential extreme values; all variables were approximately normally distributed. Paired-samples t-tests were used to compare left and right sides for muscle thickness, mean CCI and peak CCI. The effect size for paired comparisons was expressed as Cohen’s d, calculated as the mean of the paired differences divided by their standard deviation. Absolute effect sizes of about 0.20, 0.50 and 0.80 were interpreted as small, medium and large, respectively. Pearson product–moment correlation was used to examine the relationships of RMS_AP_ and RMS_ML_ in each TUG phase with the muscle thickness asymmetry index, the mean CCI asymmetry index and the peak CCI asymmetry index. Absolute correlation coefficients (r) of 0.10–0.29, 0.30–0.49 and ≥0.50 were interpreted as weak, moderate and strong, respectively. For variables that deviated from normality, contained marked outliers or violated the linearity assumption, Spearman rank correlation was planned as a sensitivity check. All variables satisfied these assumptions (Shapiro–Wilk tests, Q–Q and scatter-plot inspection), and Spearman results were consistent with Pearson results in direction and significance; therefore, only Pearson coefficients are reported. Because the study involved multiple statistical tests, the Benjamini–Hochberg (BH) procedure was applied to control the false discovery rate (FDR) within each analysis family separately. Four families were defined: (1) paired t-tests for left–right CCI differences (20 tests: 2 muscle pairs × 2 CCI measures × 5 phases); (2) paired t-tests for left–right muscle thickness differences (4 tests); (3) Pearson correlations of peak coactivation ASI with RMS (20 tests: 2 ASI variables × 2 RMS directions × 5 phases); and (4) Pearson correlations of muscle thickness ASI with RMS (40 tests: 4 ASI variables × 2 RMS directions × 5 phases). An adjusted significance level of q< 0.05 was used to identify results that survived FDR correction, and both uncorrected p-values and FDR-adjusted q-values are reported. All tests were two-tailed, and statistical significance was set at p< 0.05.

## 3 Results

**Table 1** presents the thickness of the RF, BF, TA, and MG and their ASI values (mean ± SD). Paired-samples t-tests did not reveal statistically significant left–right differences in muscle thickness for the RF (p = 0.732, d = 0.08), BF (p = 0.534, d = 0.15), TA (p = 0.495, d = 0.16), or MG (p = 0.084, d = 0.42). The corresponding thickness ASI values were 25.56% for the RF, 21.11% for the BF, 19.55% for the TA, and 16.46% for the MG. These descriptive ASI values show the magnitude of bilateral differences at the individual level, whereas the paired comparisons indicate that no consistent directional left–right difference was detected at the group level.

**Table 1:** Lower-limb muscle thickness and asymmetry index in older women (Mean ± SD, n = 27).

Variable	Left	Right	Asymmetry index (%)
RF thickness (mm)	23.13 ± 5.69	23.79 ± 6.48 [0.08]	25.56 ± 23.93
BF thickness (mm)	31.19 ± 8.07	30.09 ± 8.79 [0.15]	21.11 ± 23.89
TA thickness (mm)	26.47 ± 7.93	27.71 ± 8.29 [0.16]	19.55 ± 23.41
MG thickness (mm)	17.87 ± 4.79	19.61 ± 6.01 [0.42]	16.45 ± 16.37

Values are presented as mean ± SD for left and right muscle thickness and asymmetry index (%) of selected lower-limb muscles. Effect sizes are reported in square brackets. RF, Rectus Femoris; BF, Biceps Femoris; TA, Tibialis Anterior; MG, Medial Gastrocnemius.

**Table 2** presents the mean and peak CCI_QUAD-BF_ and CCI_TA-MG_ for the left and right sides across TUG phases, together with their asymmetry indices (mean ± SD). Paired-samples t-tests showed significant left–right differences for mean CCI_QUAD-BF_ (p< 0.001, q = 0.005, d = 0.80) and peak CCI_QUAD-BF_ (p = 0.002, q = 0.013, d = 0.65) during sit-to-stand, and for mean CCI_TA-MG_ during sit-to-stand (p = 0.001, q = 0.013, d = 0.68); all three survived BH-FDR correction. Mean CCI_QUAD-BF_ during return walk showed an uncorrected significant difference (p = 0.016, d = 0.49) but did not survive FDR correction (q = 0.077). No other left–right comparison reached significance. The descriptive ASI values for mean CCI_QUAD-BF_ were 71.78%, 39.05%, 75.96%, 56.03%, and 69.90% during sit-to-stand, outbound walk, turn, return walk, and sit-to-sit, respectively. The corresponding ASI values for mean CCI_TA-MG_ were 28.08%, 17.52%, 24.46%, 16.18%, and 28.48%, respectively. For peak CCI_QUAD-BF_, the ASI values were 18.39%, 2.76%, 32.32%, 9.96%, and 53.63% during sit-to-stand, outbound walk, turn, return walk, and sit-to-sit, respectively. The corresponding ASI values for peak CCI_TA-MG_ were 2.50%, 0.41%, 1.79%, 0.80%, and 10.03%, respectively.

**Table 2:** Mean and peak coactivation index and asymmetry index (ASI) across TUG phases in older women (Mean ± SD, n = 27).

Variable	Side	Sit-to-stand	Outbound walk	Turn	Return walk	Sit-to-sit
Mean QUAD-BF	L R	23.19 ± 13.57	35.85 ± 10.37 40.25 ± 15.38	33.40 ± 15.84 28.60 ± 17.36	34.77 ± 12.28 26.51 ± 13.31*	27.99 ± 13.73 26.53 ± 16.87
		37.34 ± 13.38*				
(%)	ASI	71.78 ± 43.35	39.05 ± 25.29	75.96 ± 54.06	56.03 ± 38.28	69.90 ± 45.32
Mean	L	42.07 ± 11.14	45.09 ± 7.72	47.29 ± 8.15	50.72 ± 9.86	46.60 ± 11.67
TA-MG (%)	R ASI	50.97 ± 9.49*	48.24 ± 7.10	45.65 ± 7.87	49.25 ± 7.47	50.03 ± 12.92
		28.08 ± 25.18	17.52 ± 13.04	24.46 ± 16.14	16.18 ± 11.82	28.48 ± 26.89
Peak QUAD-BF (%)	L	86.74 ± 17.78	98.27 ± 4.08	88.95 ± 14.69	96.62 ± 7.61	76.32 ± 18.56
	R	96.24 ± 7.04*	98.79 ± 4.11	82.27 ± 24.69	92.64 ± 12.68	68.27 ± 27.70
	ASI	18.39 ± 29.98	2.76 ± 6.53	32.32 ± 41.14	9.96 ± 16.47	53.63 ± 40.91
Peak TA-MG (%)	L	97.86 ± 5.35	99.51 ± 0.99	98.56 ± 3.67	99.27 ± 2.17	92.51 ± 13.07
	R	99.27 ± 1.00	99.57 ± 0.74	99.07 ± 0.48	99.66 ± 0.34	95.75 ± 6.21
	ASI	2.50 ± 6.23	0.41 ± 0.5	1.79 ± 4.74	0.80 ± 2.07	10.03 ± 17.93

QUAD, mean activation of Rectus Femoris, Vastus Lateralis, and Vastus Medialis; BF, Biceps Femoris; TA, Tibialis Anterior; MG, Medial Gastrocnemius. L, Left; R, Right; ASI, Asymmetry Index. *Indicates a significant left-right difference within the same condition (paired t-test, p< 0.05).

**Table 3** presents the mean and SD of RMS_AP_ and RMS_ML_ across TUG phases. **Table 4** presents the correlations of the peak CCI asymmetry indices (ASI_QUAD-BF_, ASI_TA-MG_) and the muscle thickness asymmetry indices (ASI_RF_, ASI_BF_, ASI_TA_, ASI_MG_) with RMS_AP_ and RMS_ML_ (with 95% confidence intervals provided in **Supplementary Table S2**). After BH-FDR correction within the peak coactivation ASI family, three correlations with RMS_AP_ survived: peak ASI_TA-MG_ during outbound walk (r = 0.72, p< 0.001, q< 0.001), peak ASI_QUAD-BF_ during turn (r = 0.53, p = 0.004, q = 0.043) and peak ASI_TA-MG_ during sit-to-sit (r = 0.57, p = 0.007, q = 0.044). The correlation between peak ASI_QUAD-BF_ and RMS_AP_ during outbound walk was significant before correction (r = 0.46, p = 0.016) but did not survive FDR adjustment (q = 0.081). Within the muscle thickness ASI family, ASI_MG_ showed negative correlations with RMS_AP_ during sit-to-stand (r = −0.52, p = 0.018) and outbound walk (r = −0.51, p = 0.023), but neither survived FDR correction (q = 0.461). No ASI correlated significantly with RMS_ML_. **Figure 1** presents scatter plots for the three FDR-significant correlations together with the three correlations that were significant before correction, with FDR status indicated in the panel labels. In addition, the mean CCI asymmetry indices were not significantly correlated with RMS_AP_ or RMS_ML_ in any phase (see **Supplementary Table S1**).

**Table 3:** Trunk acceleration RMS across TUG phases in older women (Mean ± SD, n = 27).

Variable	Axis	Sit-to-stand	Outbound walk	Turn	Return walk	Sit-to-sit
RMS (m s^-2^)	AP ML	1.12 ± 0.39	0.87 ± 0.28 0.68 ± 0.21	0.92 ± 0.21 0.70 ± 0.17	0.87 ± 0.16 0.70 ± 0.15	1.09 ± 0.44
		0.75 ± 0.29				0.74 ± 0.30

AP, anteroposterior axis; ML, mediolateral axis.

**Table 4:** Correlations of trunk acceleration RMS with peak coactivation asymmetry indices and muscle thickness asymmetry indices across TUG phases in older women (n = 27).

Condition	Variable	ASI_QUAD-BF_	ASI_TA-MG_	ASI_RF_	ASI_BF_	ASI_TA_	ASI_MG_
Sit-to-stand	RMS_AP_	-0.01	-0.06	0.26	0.28	0.28	-0.52*
	RMS_ML_	-0.09	0.18	-0.12	-0.08	-0.07	-0.42
Outbound walk	RMS_AP_	0.46*	0.72*†	0.03	-0.19	-0.33	-0.51*
	RMS_ML_	-0.33	-0.13	-0.21	-0.14	-0.16	-0.38
Turn	RMS_AP_	0.53*†	-0.01	-0.05	-0.28	-0.42	0.14
	RMS_ML_	0.33	0.22	-0.34	-0.21	-0.31	0.18
Return walk	RMS_AP_	0.24	0.22	-0.01	-0.16	-0.25	0.23
	RMS_ML_	-0.07	0.28	-0.37	-0.09	-0.12	0.34
Sit-to-sit	RMS_AP_	-0.16	0.57*†	0.04	0.12	0.11	0.05
	RMS_ML_	0.10	-0.26	-0.12	-0.23	-0.27	0.17

Values are Pearson correlation coefficients. RMS_AP_ and RMS_ML_ represent trunk acceleration root mean square in the anteroposterior and mediolateral directions, respectively. ASI, asymmetry index; QUAD-BF, quadriceps group versus biceps femoris; TA-MG, tibialis anterior versus medial gastrocnemius; RF, rectus femoris; BF, biceps femoris; TA, tibialis anterior; MG, medial gastrocnemius. *indicates p< 0.05 (uncorrected). † indicates q< 0.05 after BH-FDR correction within the corresponding analysis family. 95% confidence intervals for all correlation coefficients are provided in **Supplementary Table S2**.

**Figure 1:**
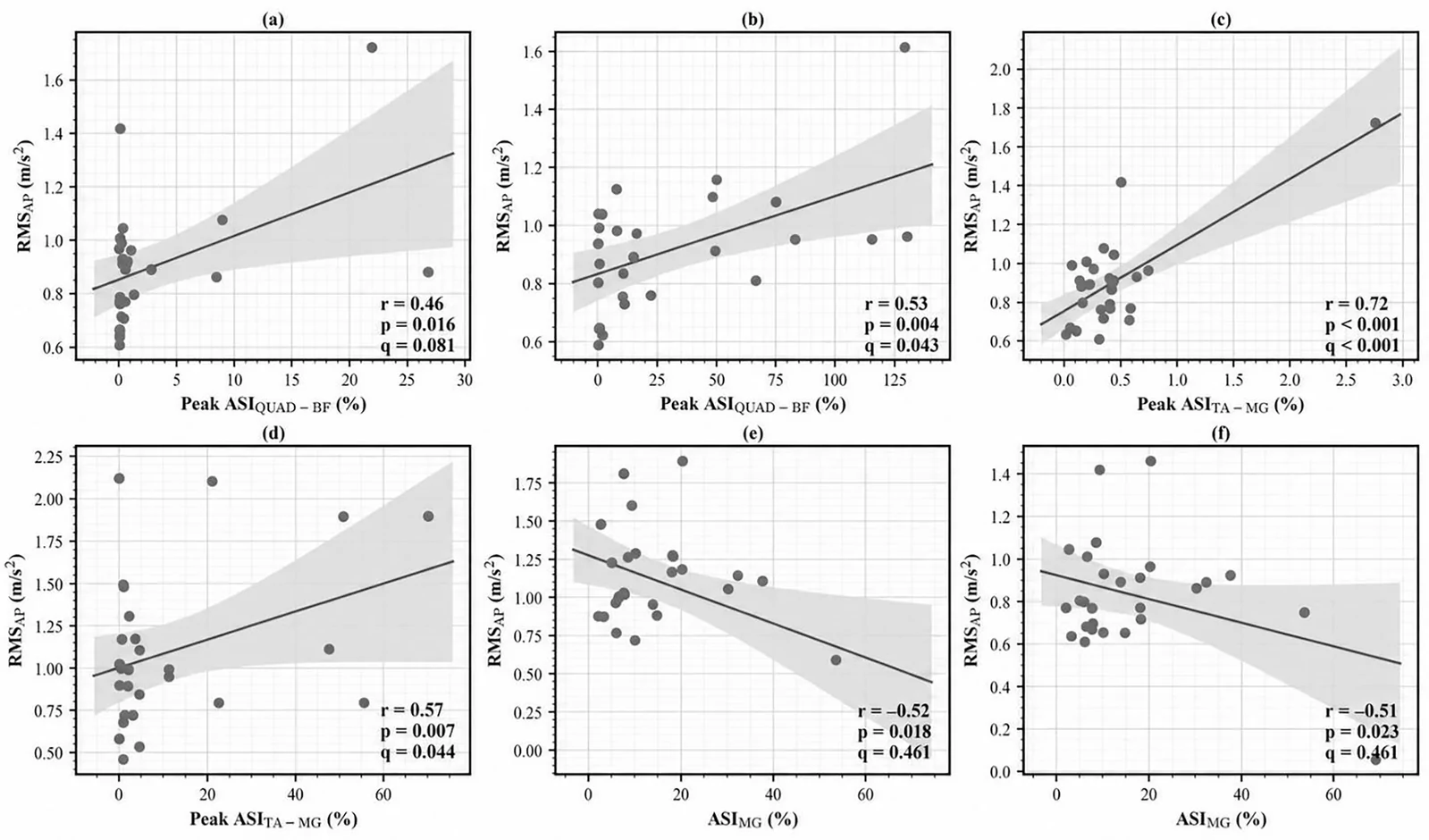
Scatter plots of the six correlations between asymmetry indices and anteroposterior trunk acceleration RMS (RMS_AP_) that reached uncorrected significance (p < 0.05). Each panel shows individual data points (gray dots), the fitted regression line and 95% confidence band; r, uncorrected p and BH-FDR-adjusted q values are shown in each panel. **(a)** Peak ASI_QUAD-BF_ vs. RMS_AP_ during outbound walk; **(b)** peak ASI_QUAD-BF_ vs. RMS_AP_ during turn; **(c)** peak ASI_TA-MG_ vs. RMS_AP_ during outbound walk; **(d)** peak ASI_TA-MG_ vs. RMS_AP_ during sit-to-sit; **(e)** ASI_MG_ vs. RMS_AP_ during sit-to-stand; **(f)** ASI_MG_ vs. RMS_AP_ during outbound walk. Panels **(b–d)** survived FDR correction (q < 0.05); panels **(a, e, f)** did not.

## 4 Discussion

First, although the thickness ASI values were >15% for all four muscles, the paired t-tests did not detect a consistent directional difference at the group level, suggesting that the direction of asymmetry was heterogeneous across individuals. Second, coactivation asymmetry at the neuromuscular level was more pronounced than morphological asymmetry, and the mean and peak measures showed distinct features: mean coactivation asymmetry was present across all phases and showed systematic left–right differences in specific phases such as sit-to-stand, whereas peak coactivation asymmetry was clearly phase-dependent. Third, anteroposterior trunk acceleration was associated mainly with coactivation asymmetry during the dynamic task rather than with static muscle thickness, and this association was concentrated in the anteroposterior direction. Taken together, lateralization of neuromuscular coordination was more consistently associated with anteroposterior trunk acceleration characteristics than resting muscle morphology. Thus, characterizing the lateralization of dynamic neuromuscular control may complement the information provided by resting muscle morphology alone.

In this study, paired t-tests did not detect statistically significant left–right differences in the mean thickness of the RF, BF, TA or MG, yet the ASI of all four muscles exceeded 15%, and the MG comparison yielded a medium effect size (d = 0.42). It is important to note that a non-significant paired t-test demonstrates only the absence of statistical evidence for a directional difference; it does not constitute evidence of bilateral equivalence. The ASI values indicate bilateral differences at the individual level, and the non-significant group means most likely reflect the heterogeneous direction of asymmetry across participants, which causes left–right differences to cancel in the group average rather than to be absent (Sadeghi et al., 2000). Mertz et al. (2019) similarly reported that healthy older adults show varying degrees of inter-limb asymmetry in muscle mass, strength and power, that the weaker side is not consistently the same limb, and that the degree of asymmetry is not linearly related to functional performance. LaRoche et al. (2012) further noted that lower-limb strength asymmetry in older women is associated with greater gait asymmetry and variability. Compared with these studies, our results indicate that similar individual heterogeneity is also present at the level of muscle thickness. Moreover, although ASI_MG_ showed uncorrected negative correlations with RMS_AP_ during sit-to-stand and outbound walk, these did not survive FDR correction, and no other thickness ASI was significantly associated with trunk acceleration RMS, suggesting that differences in resting muscle morphology may be only one of the underlying conditions affecting function and may not directly reflect dynamic control during complex tasks. Muscle thickness mainly reflects muscle volume and force-generating potential (Narici et al., 2003), whereas completion of the TUG also involves neural drive, activation timing, joint stiffness regulation and sensory feedback. Resting muscle thickness alone may therefore provide limited insight into dynamic control in older adults. The present study therefore assessed both levels to avoid attributing functional differences to a single measure.

This study found that mean coactivation asymmetry was widespread across TUG phases. Knee coactivation asymmetry was generally higher than ankle asymmetry. After FDR correction, significant left–right differences were retained for knee coactivation during sit-to-stand; the return walk difference (p = 0.016) did not survive correction (q = 0.077). The ankle showed a significant difference only during sit-to-stand. This finding is broadly consistent with previous work showing that older adults increase antagonist coactivation to enhance joint stability. Larsen et al. (2008) found that older adults showed greater lower-limb antagonist coactivation than younger adults during a stair task, which they interpreted as a compensatory strategy for declining strength and impaired postural control. Acuña et al. (2019) likewise found that older adults increase lower-limb antagonist coactivation to enhance joint stability when sensory or cognitive load is raised. However, previous studies have focused mainly on coactivation in a single limb, whereas the present study further shows that, even when overall coactivation increases, its distribution between the left and right limbs may be uneven. According to Latash’s (2018) account of the functions of antagonist coactivation, coactivation increases the apparent stiffness of a joint and reduces its degrees of freedom, but also raises energy cost and may limit rapid postural adjustment. The high mean coactivation ASI may indicate that, in some participants, one limb contributed disproportionately to joint-stabilizing muscular activity. Such lateralization may be an adaptive strategy to maintain task completion or may reflect a decline in bilateral neuromuscular coordination; the present cross-sectional data cannot distinguish between these possibilities.

Mean and peak coactivation differed in their phase profiles, suggesting that they capture different aspects of neuromuscular control—a distinction that most previous studies, reporting only a single coactivation value, have been unable to make. The mean CCI describes the average level of sustained antagonist overlap throughout a phase, whereas the peak CCI reflects how closely the two muscle groups are activated at a single brief moment. Peak knee coactivation asymmetry was low during the outbound and return walk but rose markedly during the turn and sit-to-sit, indicating that instantaneous coactivation asymmetry is not constant but becomes more pronounced when movement demands change rapidly. Using the instrumented TUG, Salarian et al. (2010); Zampieri et al. (2010) and Weiss et al. (2010) found that standing up, straight-line walking, turning and sitting down have distinct kinematic characteristics, and that phase-specific measures can reveal differences in motor control that total TUG time cannot. Our results are consistent with this view and extend phase specificity to the level of bilateral neuromuscular coordination. Standing up requires the rapid generation of forward momentum and the completion of knee and hip extension; turning requires the medial and lateral limbs to take on different braking and supporting roles; and sitting down requires control of forward momentum while lowering the body’s center of mass. All of these non-steady phases may amplify differences in bilateral control. It should be noted that peak ankle CCI was close to 100% in most phases, with a correspondingly low ASI. This may indicate that the ankle antagonists are relatively symmetrical at the moment of maximal overlap, or it may relate to the computational properties of the coactivation index: when the normalized amplitudes of the two muscle groups are similar, the index approaches its upper limit and reflects only the relative overlap of activation rather than the absolute amount (Gagnat et al., 2020). Peak CCI should therefore not be equated directly with the absolute level of muscle activation or with joint mechanical stiffness.

By recording bilateral sEMG and L4 trunk acceleration simultaneously and dividing the TUG into five phases, this study was able to analyze the phase-specific relationship between asymmetry and trunk acceleration. After FDR correction, peak ankle coactivation asymmetry during outbound walk and sit-to-sit, and peak knee coactivation asymmetry during turn, remained significantly positively correlated with RMS_AP_; the correlation between peak knee coactivation asymmetry and RMS_AP_ during outbound walk did not survive correction, whereas none of the correlations with RMS_ML_ reached significance. Menz et al. (2003) noted that trunk acceleration reflects the smoothness of center-of-mass motion and features of postural control during walking, and that acceleration in different directions has different functional meaning. Anteroposterior acceleration is more readily affected by propulsion, braking and changes in speed, whereas mediolateral stability depends mainly on step-by-step foot placement and lateral balance control (MacKinnon and Winter, 1993). Initiation of the outbound walk, deceleration during turning and braking when sitting down in the TUG all involve rapid adjustments of forward momentum. Bilateral differences in peak coactivation may therefore be associated with greater anteroposterior trunk acceleration, although whether this reflects an imbalance in propulsion and braking forces cannot be confirmed without kinetic data. However, because a higher RMS_AP_ can also arise from greater movement intensity or acceleration demands rather than from poorer control per se, this association should be interpreted as indicating that coactivation asymmetry is related to anteroposterior acceleration characteristics during the TUG, rather than as direct evidence of reduced stability. This finding partly supports the view of Hill and Nantel (2019) that lower-limb asymmetry can reduce dynamic trunk stability. However, no significant correlation was found between coactivation ASI and RMS_ML_. Plotnik et al. (2007) noted that bilateral gait coordination is a relatively independent feature of gait that deteriorates with ageing and Parkinson’s disease; the lack of a significant bilateral association for mediolateral acceleration in our study may be related to the nature of the task. Previous studies have mostly analyzed continuous steady-state walking, whereas the TUG comprises several short non-steady phases. In addition, all participants in this study walked independently and had no self-reported balance disorder, so between-individual variation in RMS_ML_ may have been small. Our results are therefore more likely to reflect phase-related changes in propulsion and braking control during the TUG, and cannot be taken to rule out a potential relationship between coactivation asymmetry and mediolateral stability. Overall, phase-specific neuromuscular measures may provide additional descriptive information beyond total TUG time. Non-steady phases such as sit-to-stand, turning, and sitting down may be particularly informative for characterizing bilateral coordination differences.

The negative correlations between ASI_MG_ and RMS_AP_ during sit-to-stand and outbound walk did not survive FDR correction and should be regarded as exploratory. The general assumption is that greater muscle morphological asymmetry would be accompanied by less balanced motor control and greater trunk sway, yet our results showed the opposite direction. One possible explanation relates to the role of the medial gastrocnemius in propulsion: the medial gastrocnemius is a key ankle plantarflexor that provides propulsive power in late stance (Farris and Sawicki, 2012). Previous work has shown that, when there is strength or morphological asymmetry between the lower limbs, older adults tend to rely more on the stronger side for support and propulsion, maintaining walking performance through a redistribution of bilateral load (Gurney et al., 2001; Si et al., 2024). Some participants may have relied more on the side with greater gastrocnemius reserve for propulsion, temporarily reducing anteroposterior trunk acceleration during sit-to-stand and initial walking. This proposed compensatory mechanism remains speculative and requires verification with bilateral kinetic data. Given that the thickness ASI showed no stable association in other phases or directions, this negative correlation should be regarded as an exploratory finding rather than evidence that muscle morphological asymmetry is protective. Future studies should combine bilateral joint kinetics and plantar pressure to test the robustness of this result.

This study has several limitations. First, the cross-sectional design can establish associations only and cannot determine whether coactivation asymmetry is a cause of altered trunk acceleration or a compensatory response. Second, only healthy community-dwelling older women were included, so the results cannot be generalized directly to older men, fallers, or people with neuromuscular disease. Third, the TUG phase segmentation was based on instrumented-TUG approaches reported by Salarian et al. (2010); Weiss et al. (2010), and Zampieri et al. (2010) and was visually checked trial by trial. However, the specific five-phase rule-based implementation used in the present study was adapted from these approaches and was not independently validated against three-dimensional motion capture or force-plate data. Since the main findings are phase-specific, segmentation error may have affected the phase-level results, although visual inspection was used to reduce the risk of gross mis-segmentation. Fourth, reliability information for the phase-specific outcomes was limited because phase-specific ICCs with confidence intervals were not calculated for the CCI, ASI, RMS_AP_, or RMS_ML_ outcomes. Although CVs were used to assess between-trial consistency for selected phase-specific measures and ICCs were available for RMS_AP_ and RMS_ML_ across the complete TUG trials, the test–retest reliability of individual phase-specific outcomes was not fully characterized. Fifth, the sample size (n = 27) exceeded the *a priori* requirement for detecting a single correlation of r = 0.50 but was not powered for the number of comparisons conducted; although BH-FDR correction was applied, some associations may still reflect chance findings. Sixth, the CCI reflects the relative overlap of normalized EMG envelopes rather than joint torque or mechanical stiffness, so the bilateral differences reported here describe asymmetry in activation patterns rather than in joint stability per se. Seventh, limb dominance was not formally assessed, limiting the ability to distinguish anatomical asymmetry from functional lateralization. Future studies should validate the phase-segmentation procedure against a gold-standard reference system, further evaluate the reliability of phase-specific CCI and ASI outcomes using ICCs with confidence intervals, enlarge the sample, and include bilateral kinetic measures and formal assessment of limb dominance.

## 5 Conclusion

This study examined the association between lower-limb asymmetry and trunk acceleration during TUG functional mobility in healthy community-dwelling older women at two levels, muscle morphology and neuromuscular control. The four lower-limb muscles showed no significant group-level left–right thickness differences, but individual-level asymmetry was common, confirming that the direction of asymmetry is heterogeneous across participants. Coactivation asymmetry was widespread across TUG phases and phase-dependent, with systematic left–right differences in knee and ankle coactivation during sit-to-stand (all three comparisons surviving FDR correction). Compared with resting muscle thickness, peak coactivation asymmetry in specific phases showed a more consistent association with RMS_AP_, suggesting that dynamic neuromuscular coordination may offer additional insight into anteroposterior acceleration characteristics during functional tasks in older adults. These findings suggest that, for the assessment of mobility function in older adults, resting muscle thickness or total TUG time alone may not be sufficient to capture potential bilateral coordination problems, whereas phase-specific neuromuscular measures during a dynamic task showed a more consistent association with anteroposterior trunk acceleration in the present sample. Among these, non-steady phases such as sit-to-stand, turning and sitting down may deserve particular attention, and RMS_AP_ could be explored as a candidate measure for accelerometer-based monitoring. Because bilateral control differences were exposed mainly during non-steady phases such as sit-to-stand, turning and sitting down, future work should investigate whether training programs targeting these movements can improve bilateral coordination and functional mobility. These findings suggest that phase-specific coactivation asymmetry is associated with anteroposterior trunk acceleration characteristics during the TUG. Further studies are required to establish the reliability, validity, and clinical relevance of these phase-specific measures before they can be considered for broader functional assessment.

## Data Availability

The raw data supporting the conclusions of this article will be made available by the authors, without undue reservation.
