# Supplementary material for: Bilateral lower-limb asymmetry and phase-specific trunk acceleration during the Timed Up and Go in older women: an analysis based on muscle morphology and antagonist coactivation
Source: Front Physiol. 2026 Oct 2;17:1915589. doi: 10.3389/fphys.2026.1915589 (PMC13671921; doi:10.3389/fphys.2026.1915589)
Supplement: Supplementary file 1 [file SupplementaryFile1.docx]

Supplementary Material

# Supplementary Tables S1

Supplementary Table S1. Correlations of trunk acceleration RMS with mean coactivation asymmetry indices across TUG phases in older women (n = 27).

| Condition | Variable | ASI_QUAD-BF_ | ASI_TA-MG_ |
| --- | --- | --- | --- |
| Sit-to-stand | RMS_AP_ | -0.01 (p=0.969) | -0.09 (p=0.678) |
|  | RMS_ML_ | -0.10 (p=0.615) | -0.03 (p=0.882) |
| Outbound walk | RMS_AP_ | 0.11 (p=0.585) | 0.14 (p=0.477) |
|  | RMS_ML_ | 0.12 (p=0.538) | -0.06 (p=0.768) |
| Turn | RMS_AP_ | 0.39 (p=0.056) | -0.04 (p=0.862) |
|  | RMS_ML_ | 0.35 (p=0.070) | 0.00 (p=0.990) |
| Return walk | RMS_AP_ | 0.14 (p=0.495) | 0.17 (p=0.399) |
|  | RMS_ML_ | 0.28 (p=0.156) | 0.32 (p=0.098) |
| Sit-to-sit | RMS_AP_ | -0.05 (p=0.830) | 0.29 (p=0.205) |
|  | RMS_ML_ | -0.01 (p=0.972) | -0.40 (p=0.074) |

Note: Values are Pearson correlation coefficients. RMS_AP_ and RMS_ML_ represent trunk acceleration root mean square in the anteroposterior and mediolateral directions, respectively. ASI = asymmetry index; QUAD-BF = quadriceps group (mean of rectus femoris, vastus lateralis, and vastus medialis) versus biceps femoris; TA-MG = tibialis anterior versus medial gastrocnemius. * indicates p < 0.05. No correlation reached statistical significance.

# Supplementary Table S2

Pearson correlation coefficients (r) with 95% confidence intervals between asymmetry indices and trunk acceleration RMS across TUG phases (n = 27).

(a) Peak coactivation asymmetry index

| TUG phase | Variable | Peak ASI_QUAD-BF_ | Peak ASI_TA-MG_ |
| --- | --- | --- | --- |
| Sit-to-stand | RMSAP | -0.01 [-0.40, 0.38] | -0.06 [-0.44, 0.34] |
|  | RMSML | -0.09 [-0.46, 0.31] | 0.18 [-0.23, 0.53] |
| Outbound walk | RMSAP | 0.46 [0.10, 0.71]* | 0.72 [0.47, 0.87]† |
|  | RMSML | -0.33 [-0.63, 0.06] | -0.13 [-0.49, 0.26] |
| Turn | RMSAP | 0.53 [0.19, 0.76]† | -0.01 [-0.39, 0.37] |
|  | RMSML | 0.33 [-0.06, 0.63] | 0.22 [-0.18, 0.55] |
| Return walk | RMSAP | 0.24 [-0.15, 0.57] | 0.22 [-0.17, 0.56] |
|  | RMSML | -0.07 [-0.44, 0.32] | 0.28 [-0.11, 0.60] |
| Sit-to-sit | RMSAP | -0.16 [-0.55, 0.30] | 0.57 [0.19, 0.81]† |
|  | RMSML | 0.10 [-0.34, 0.51] | -0.26 [-0.62, 0.20] |

Values are presented as r [95% CI]. * p < 0.05 (uncorrected); † q < 0.05 (Benjamini–Hochberg FDR corrected). Exact p-values and q-values for the marked correlations are shown in Figure 1.

(b) Muscle thickness asymmetry index

| TUG phase | Variable | ASI_RF_ | ASI_BF_ | ASI_TA_ | ASI_MG_ |
| --- | --- | --- | --- | --- | --- |
| Sit-to-stand | RMS_AP_ | 0.26 [-0.20, 0.63] | 0.28 [-0.19, 0.64] | 0.28 [-0.19, 0.64] | -0.52 [-0.78, -0.11]**^*^** |
|  | RMS_ML_ | -0.12 [-0.54, 0.34] | -0.08 [-0.51, 0.37] | -0.07 [-0.50, 0.39] | -0.42 [-0.73, 0.03] |
| Outbound walk | RMS_AP_ | 0.03 [-0.42, 0.47] | -0.19 [-0.58, 0.28] | -0.33 [-0.67, 0.13] | -0.51 [-0.77, -0.08]**^*^** |
|  | RMS_ML_ | -0.21 [-0.59, 0.26] | -0.14 [-0.55, 0.32] | -0.16 [-0.56, 0.31] | -0.38 [-0.70, 0.07] |
| Turn | RMS_AP_ | -0.05 [-0.49, 0.40] | -0.28 [-0.64, 0.19] | -0.42 [-0.73, 0.03] | 0.14 [-0.33, 0.55] |
|  | RMS_ML_ | -0.34 [-0.68, 0.12] | -0.21 [-0.59, 0.26] | -0.31 [-0.66, 0.15] | 0.18 [-0.29, 0.58] |
| Return walk | RMS_AP_ | -0.01 [-0.45, 0.44] | -0.16 [-0.56, 0.30] | -0.25 [-0.62, 0.22] | 0.23 [-0.23, 0.61] |
|  | RMS_ML_ | -0.37 [-0.70, 0.09] | -0.09 [-0.51, 0.36] | -0.12 [-0.53, 0.34] | 0.34 [-0.12, 0.68] |
| Sit-to-sit | RMS_AP_ | 0.04 [-0.43, 0.48] | 0.12 [-0.36, 0.54] | 0.11 [-0.36, 0.54] | 0.05 [-0.42, 0.49] |
|  | RMS_ML_ | -0.12 [-0.55, 0.35] | -0.23 [-0.62, 0.25] | -0.27 [-0.65, 0.21] | 0.17 [-0.30, 0.58] |

Values are presented as r [95% CI]. * p < 0.05 (uncorrected); † q < 0.05 (Benjamini–Hochberg FDR corrected). Exact p-values and q-values for the marked correlations are shown in Figure 1.

Peak AS_IQUAD-BF_ and Peak ASI_TA-MG_ = peak coactivation asymmetry index of the quadriceps–biceps femoris and tibialis anterior–medial gastrocnemius muscle pairs, respectively. ASI_RF_, ASI_BF_, ASI_TA_, ASI_MG_ = muscle thickness asymmetry index of the rectus femoris, biceps femoris, tibialis anterior and medial gastrocnemius, respectively.
